# Cellular senescence in diabetic retinopathy: Molecular mechanisms and therapeutic targeting

**DOI:** 10.1016/j.aopr.2026.07.002

**Published:** 2026-08-29

**Authors:** Wenhui Liu, Jingzhao Gao, Xinyi Zhang, Fuhua Yang, Mingfei Jiao, Jin Chang, Lin Su, Xiaorong Li

**Affiliations:** a Tianjin Key Laboratory of Retinal Functions and Diseases, Tianjin Branch of National Clinical Research Center for Ocular Disease, Eye Institute and School of Optometry, Tianjin Medical University Eye Hospital, Tianjin, China; b Medical College, Inner Mongolia Minzu University, Tongliao, China; c School of Life Sciences, Tianjin University, Tianjin, China

**Keywords:** Diabetic retinopathy, Cell senescence, Neurovascular unit, Oxidative stress, Senescence-associated secretory phenotype

## Abstract

**Background:**

Diabetic retinopathy (DR) is the leading cause of vision loss in the working-age population, traditionally attributed to metabolic disorders and microvascular damage induced by chronic hyperglycemia. Emerging evidence indicates that cellular senescence acts as a critical upstream driver of DR, with senescent cells accumulating in the retina and secreting a senescence-associated secretory phenotype (SASP) factors that promote chronic inflammation, pathological angiogenesis, and disruption of the blood-retinal barrier (BRB).

**Main text:**

This review adopts a neurovascular unit (NVU) perspective to synthesize cell-type-specific senescence phenotypes in the diabetic retina, emphasizing that endothelial cell, pericyte, retinal pigment epithelium, microglial, and retinal ganglion cell senescence constitute an interconnected pathological network rather than isolated events. NVU-wide senescence establishes a self-reinforcing cycle: senescent endothelial and pericyte dysfunction drives vascular instability; retinal pigment epithelial cells senescence compromises the outer BRB; and glial cell and retinal ganglion cell senescence amplifies neuroinflammation and neurodegeneration. We dissect key molecular drivers—oxidative DNA damage, epigenetic reprogramming (miRNA/Sirtuins), mitophagy impairment, and SASP-mediated inflammatory feedforward—and critically evaluate emerging senotherapeutics (senolytics, senomorphics, and precision delivery platforms) within an evidence-graded framework.

**Conclusions:**

Cellular senescence establishes a progressive senescence-inflammation vicious cycle in DR, affecting multiple interconnected cell types within the NVU and causing neurovascular dysfunction. Targeting cellular senescence through senescent cell clearance, SASP inhibition, mitochondrial homeostasis regulation, and epigenetic modulation offers promising new therapeutic directions. With the advancement of novel senotherapeutics and precision technologies, personalized early treatment and retinal repair for DR may become achievable in the future.

## 1 Introduction

Diabetic retinopathy (DR) is the most common ocular complication of diabetes and has become the leading cause of visual impairment in the working-age population worldwide.^1^ Traditionally, it has been believed that metabolic disorders, oxidative stress, and damage to retinal microvascular structures due to prolonged hyperglycemia are the primary pathological factors driving the onset and progression of DR.^2^^,^^3^ However, recent basic and clinical studies have increasingly suggested that cellular senescence may be a more upstream, multi-step common mechanism behind these classical pathological changes. Animal experiments and human retinal tissue studies have revealed abnormal accumulation of senescent cells in the retinas of patients with diabetes and proliferative DR. The senescence-associated secretory phenotype (SASP) factors released by these cells can induce chronic inflammation, promote pathological angiogenesis, and disrupt extracellular matrix homeostasis, ultimately accelerating structural damage and functional impairment of the retinal neurovascular unit (NVU).^4^, ^5^, ^6^, ^7^, ^8^, ^9^, ^10^ These findings suggest that cellular senescence is not only the consequence of chronic diabetic injury but may also serve as a key initiator and amplifier of the pathological cascade in DR.^11^

In this review, we first summarize clinical and experimental evidence supporting the involvement of cellular senescence in DR. We then discuss cell-type-specific senescence phenotypes in the diabetic retina, dissect the underlying molecular mechanisms, and finally highlight senescence-targeted therapeutic strategies and future directions. In recent years, several reviews have summarized the markers and therapeutic potential of cellular senescence in DR.^4^^,^^7^^,^^12^ Moreover, preclinical and phase I studies have preliminarily validated the feasibility of targeting senescence in diabetic macular edema (DME).^9^ However, existing literature has largely focused on single cell types or isolated mechanisms, lacking a holistic analysis of the reciprocal crosstalk among diverse senescent cell populations within the NVU and the paracrine propagation of senescence signals. Moreover, the key obstacles hindering the clinical translation of targeted therapies have not been thoroughly explored. The following features distinguish this review: (1) To systematically integrate the aging phenotypes of retinal cell types and their synergistic damage effects in NVU. (2) The cross-regulatory network of the mitochondrial reactive oxygen species (ROS)-mtDNA/cGAS-STING-SASP positive feedback loop and epigenetic remodeling was analyzed. (3) Based on the experiment-transformation-clinical evidence grading framework, the transformation bottleneck of anti-aging drugs was evaluated, and new strategies for precise delivery and combined intervention were proposed.

Given that the evidence covered in this review derives from multiple research layers with substantially different levels of credibility and translational potential, we employ descriptive language throughout the text to distinguish the sources of evidence for key conclusions. Specifically, we use terms such as clinical studies or in patients with DR to denote human clinical evidence, ex vivo human tissues for human tissue-based studies, animal models or preclinical studies for in vivo experimental evidence, and in vitro experiments or mechanistic studies for cell-based mechanistic investigations. This stratified terminology is intended to help readers clearly identify the evidence level of each conclusion and to objectively assess the current gap between basic research and clinical translation.

## 2 Evidence for cellular senescence in diabetic retinopathy

Unlike aging, cellular senescence is an irreversible state of cell cycle arrest that occurs at the cellular level when cells experience various forms of stress. Typical features of senescent cells include increased senescence-associated β-galactosidase (SA-β-gal) activity, upregulation of the p16^INK4a^/p21 signaling pathway, and the secretion of a variety of SASP factors. In recent years, clinical studies have shown that the levels of senescence markers, such as p16^INK4a^ and SASP factors, are significantly increased in the retinas of patients with DR and DME. Their expression is positively correlated with pathological changes, including microaneurysms and the enlargement of capillary non-perfusion areas.^7^, ^8^, ^9^^,^^12^^,^^13^ Animal studies further confirmed that in db/db mice and the oxygen-induced retinopathy (OIR) model, the number of senescence marker-positive cells was increased, and the expression of senescence markers exhibited spatiotemporal concordance with pathological neovascularization.^5^^,^^8^^,^^14^ In vitro experiments have demonstrated that under persistent hyperglycemic conditions, retinal endothelial cells (ECs), pericytes and retinal pigment epithelium (RPE) cells exhibit significant premature senescence, accompanied by continuous SASP release. This in turn induces chronic inflammation, increases vascular permeability, and promotes pathological neovascularization.^15^, ^16^, ^17^, ^18^, ^19^ Multiple mechanistic studies have further implicated oxidative stress, DNA damage, epigenetic regulation abnormalities, and mitochondrial dysfunction in this process. These pathways collectively drive the multi-dimensional pathological process of DR.^5^^,^^7^, ^8^, ^9^ Collectively, these observations support the notion that cellular senescence is not merely a bystander but a driver of DR pathology.

## 3 Cell-type-specific senescence phenotypes and neurovascular unit dysfunction in the diabetic retina

### 3.1 Endothelial cell senescence and breakdown of the inner BRB

Retinal vascular ECs are the core components of the blood-retinal barrier (BRB), and their structural and functional impairment is a key pathological basis of DR.^5^^,^^9^ As shown in Fig. 1, in vitro experiments have shown that hyperglycemia promotes ECs to enter a senescence state,^17^^,^^20^ characterized by elevated SA-β-gal activity, reduced proliferation ability and increased secretion of SASP factors.^13^^,^^17^^,^^21^ At the mechanistic level, in vitro studies have revealed that in a hyperglycemic microenvironment, ROS accumulate significantly in ECs, triggering a DNA damage response and disrupting telomere homeostasis. This, in turn, activates the p53/p21 and p16/Rb pathways, promoting ECs to enter the senescence process^16^^,^^17^ (shown in Fig. 1). Simultaneously, upregulation of Nox4 expression is an important source of oxidative stress in diabetic retinal ECs. Animal studies have shown that Nox4 is significantly increased in DR mice, where it induces EC senescence, leading to pericyte loss and exacerbation of vascular damage.^22^ Hypoxic and inflammatory environments can further reinforce the senescent phenotype in ECs. In the OIR model, the expression of *Cdkn2a*/p16^INK4a^ and various SASP factors, such as plasminogen activator inhibitor-1 (PAI-1) and vascular endothelial growth factor (VEGF), peaked simultaneously with the onset of pathological neovascularization, accompanied by a large number of SA-β-gal-positive regions.^5^^,^^8^ Additionally, the downregulation of the sirtuin family represents another key pro-senescence mechanism.^23^, ^24^, ^25^, ^26^, ^27^, ^28^ Innate immune signaling pathways are also implicated in driving EC senescence. In vitro studies have demonstrated that hyperglycemia activates the cGAS-STING pathway, impairing mitophagy; mtDNA leakage further amplifies this activation, ultimately promoting inflammation and the release of SASP factors.^29^, ^30^, ^31^ Single-cell RNA sequencing in db/db mice further revealed that p53 plays a central role in regulating EC senescence, with its expression level directly influencing whether ECs enter the senescence state.^14^ These findings suggest a close relationship between EC senescence and aberrant angiogenesis, which eventually leads to BRB damage, increased vascular permeability and pathological neovascularization. Beyond its direct effects on vascular integrity, endothelial cell senescence exerts broader pathophysiological consequences through paracrine signaling. It is noteworthy that the impact of endothelial cell senescence is not confined to the vascular wall itself. SASP factors released by senescent ECs, such as endothelin-1 (ET-1), tumor necrosis factor (TNF)-α, and VEGF, can act on adjacent pericytes via paracrine pathways, leading to reduced pericyte coverage and compromised vascular stability.^5^^,^^25^ Simultaneously, these inflammatory factors can diffuse into the nerve fiber layer, directly damaging axonal mitochondria in retinal ganglion cells (RGCs) and inducing synaptic degeneration.^32^ These findings suggest that vascular senescence signals can propagate through the microenvironment and exacerbate neurodegeneration, implying that the relationship between vascular and neural pathology in DR is not a simple linear sequence, but rather a mutually reinforcing vicious cycle driven by senescence. The above conclusions regarding endothelial cell senescence are primarily supported by in vitro and animal model evidence, with direct validation in DR patients awaiting further clinical investigation.

**Fig. 1:**
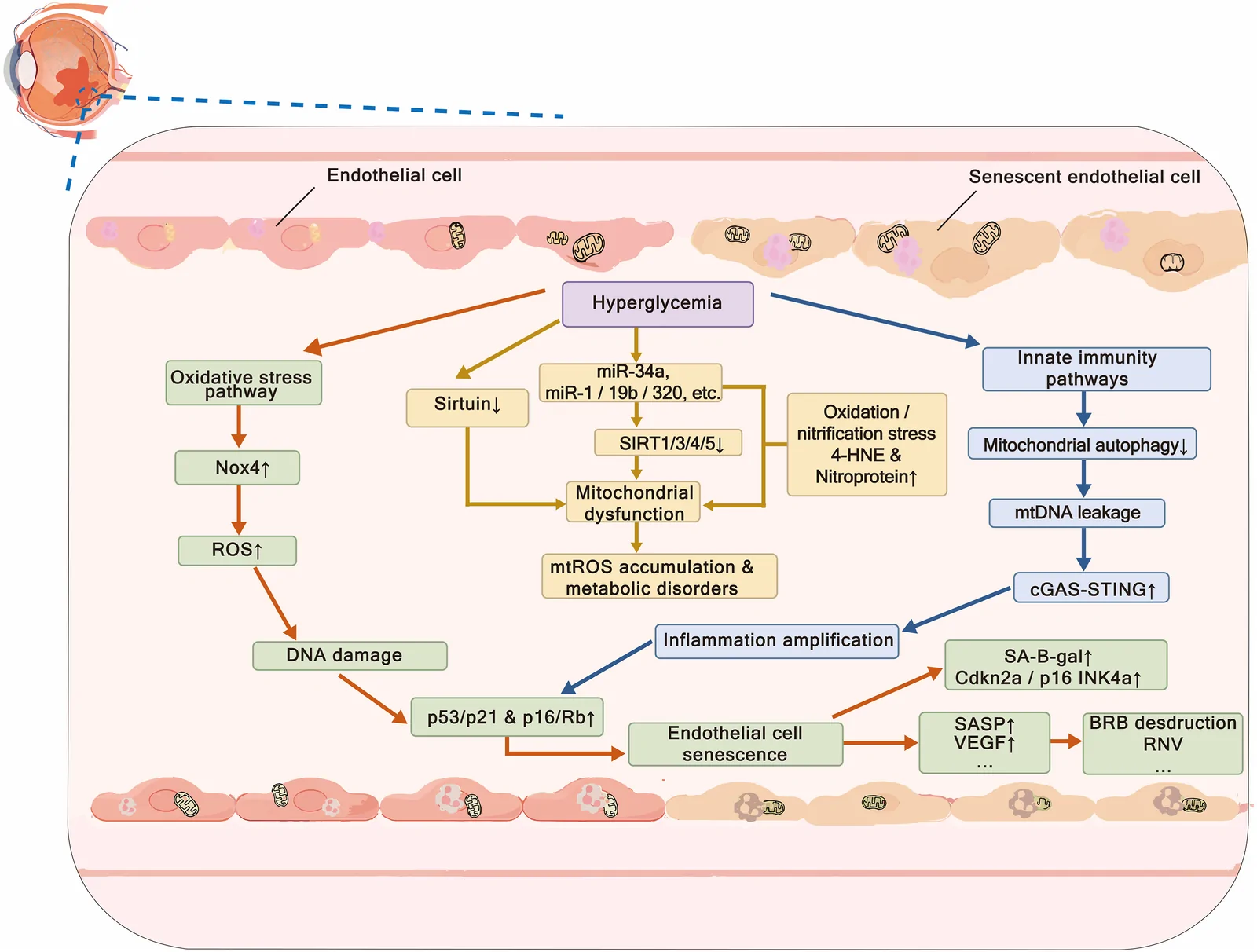
Key factors inducing endothelial cell senescence in diabetic retinopathy. The figure above illustrates a series of mechanisms through which hyperglycemia induces endothelial cell senescence. Among these, increased oxidative stress, increased ROS and decreased sirtuin family and mitochondrial autophagy are the focus of this section's discussion, and these changes are also simultaneously reflected in the endothelial DNA damage and inflammation amplification. Under hyperglycemic conditions, the above factors lead to the up-regulation of p53/p21 & p16/Rb and endothelial cell senescence, accompanied by the expression of senescence markers such as SA-B-gal and *Cdkn2a*/p16 ^INK4a^ and the up-regulation of pro-angiogenic factors such as SASP and VEGF, eventually leading to BRB destruction and RNV.

### 3.2 Pericyte senescence and early microvascular instability

Pericytes are essential structural components of the BRB, maintaining capillary stability through physical adhesion and paracrine signaling with ECs. Traditionally, pericyte dropout is considered one of the earliest vascular lesions in DR, leading to the loss of structural support for ECs. In vitro studies have demonstrated that pericyte senescence occurs under hyperglycemic conditions, with an increase in the secretion of SASP factors. This not only weakens pericyte support for ECs but also exacerbates the inflammatory response, revealing a continuous pathological process that progresses from “functional inactivation to structural destruction”.^33^^,^^34^ Clinical studies have shown that the expression level of transforming growth factor (TGF)-β1 in plasma is significantly elevated in diabetic patients. In vitro experiments further confirmed that TGF-β1 induces primary pericytes to enter a senescent state, accompanied by a significant increase in SASP secretion, particularly the upregulation of VEGF and interleukin (IL-6).^35^ These findings suggest that hyperglycemia-driven circulating inflammation and local senescence act synergistically to amplify the pathological process. Single-cell RNA sequencing has further supported this view: in the DR model, pericytes were enriched with numerous senescence-related transcripts, with significant upregulation of *Cdkn2a*/p16^*INK4a*^, *Cdkn1a*/p21, *Serpine1*/PAI-1, *Vegfa*, and *Tgfb1*.^5^ Animal studies have shown that senescent pericytes can recruit and activate neutrophils through SASP factors, such as IL-1β and C-X-C motif chemokine ligand 1 (CXCL1), leading to the formation of focal neutrophil extracellular traps (NETs), which further amplify local inflammation and tissue damage.^8^ Therefore, pericyte senescence leads to decreased EC support function and the formation of microaneurysms and capillary occlusion. Beyond their own diminished supportive capacity, senescent pericytes secrete IL-1β and CXCL1, which can recruit neutrophils to form NETs, further exacerbating local microthrombosis and inflammatory injury.^8^ Concomitantly, the dysregulated expression of TGF-β1 and VEGF by senescent pericytes not only compromises pro-survival signaling to ECs—such as the PDGF-BB/PDGFR-β axis—but also induces secondary dysfunction in adjacent ECs.^35^ This cascade, whereby pericyte senescence drives endothelial destabilization and thereby exacerbates vascular leakage, reveals the central hub role of pericytes in initiating early BRB breakdown. Evidence in this area is predominantly derived from in vitro and animal model studies, with direct evidence from DR patients still awaiting further accumulated.

### 3.3 Senescence of retinal pigment epithelium and outer BRB dysfunction

RPE plays a critical role in maintaining the homeostasis of the outer BRB, visual circulation and photoreceptor metabolism.^36^ In DR, prolonged hyperglycemia, oxidative stress, lipid metabolism disorders, and inflammation can induce stress-induced premature senescence of RPE. ^18^^,^^19^^,^^37^, ^38^, ^39^, ^40^ In vitro studies have shown that senescent RPE cells exhibit significant downregulation of key tight junction proteins (ZO-1, occludin) and adherens junction proteins (E-cadherin), compromising the integrity of the outer BRB and increasing its permeability. This, in turn, facilitates the infiltration of harmful molecules into deeper retinal layers, exacerbating the inflammatory cascade.^36^^,^^41^ In vitro experiments have demonstrated that short-term or medium-term hyperglycemia exposure (10–50 mM for 24–72 h) induces a SASP phenotype in ARPE-19 cells, accompanied by down-regulation of SIRT1 and activation of the PI3K/Akt/mTOR signaling axis, which further promotes apoptosis of RPE cells.^18^^,^^38^^,^^39^ In vitro studies have further revealed that hyperglycemia also upregulates the mitochondrial anchor protein TIN2, activates mTOR, inhibits mitophagy, causes mtDNA damage and accumulation of mitochondrial ROS (mtROS), ultimately exacerbating the RPE senescent phenotype.^37^ Moreover, due to the high susceptibility of RPE to oxidative stress, in vitro experiments have shown that exposure to a low concentration of 50 μM H_2_O_2_ for just 30 min is enough to trigger senescence, primarily by activating the cGAS–STING–NF-κB/HIF-1α axis, which in turn increases VEGF expression and senescence.^40^ This indicates the formation of a positive feedback loop between oxidative damage, inflammatory signaling and senescence. In conclusion, RPE senescence leads to the destruction of the outer BRB and promotes the diffusion of inflammatory cytokines and harmful metabolites to the inner layer. In addition to causing breakdown of the outer BRB, senescent RPE cells secrete abundant VEGF, IL-6, and MMP-2/9, which can diffuse into the inner retina, disrupt the extracellular matrix microenvironment of photoreceptors, and induce inflammatory transformation of Müller cells, thereby further amplifying the glial response.^36^^,^^41^ Given the location of the RPE at the interface between the choroid and the neural retina, the “outer-to-inner” diffusion trajectory of its senescence-associated secretory products may constitute a critical upstream driver of photoreceptor dysfunction and late-stage macular edema in DR. Current evidence for RPE senescence is derived almost exclusively from in vitro cell experiments, with validation in animal models and clinical samples remaining insufficient. Future translational studies are urgently needed.

### 3.4 Senescence of microglia, astrocytes and infiltrating macrophages

Neuroglial cells and immune cells in the retina also undergo senescence in the diabetic condition, amplifying inflammation, metabolic disturbances and tissue damage. Microglia, the resident immune cells of the retina, are involved in inflammatory surveillance, synaptic pruning, and the regulation of cell survival.^42^ In diabetic conditions, microglia not only become activated but also exhibit senescence-related transcriptional and functional changes. In the OIR model, microglia are significantly activated at the peak of the disease course, and their activation parallels the secretion of SASP factors.^43^ In vitro studies have shown that PPARα-deficient microglia release large amounts of extracellular vesicles (EVs) in a hyperglycemic environment. These EVs contain pro-inflammatory and pro-senescence molecules that act on astrocytes, inducing their senescence phenotype, characterized by upregulation of p16/p21 and enhanced SASP secretion. This process ultimately disrupts the neuroglial network and contributes to retinal dysfunction.^44^

Under pathological conditions, macrophages can be recruited from the systemic circulation into the retina, where they may persist within the local tissue.^45^^,^^46^ In the diabetic inflammatory microenvironment, these macrophages also undergo senescence, which alters their immunoregulatory functions. In vitro experiments have demonstrated that the lipid peroxidation product 4-Hydroxynonenal induces a distinct senescent phenotype in RAW264.7 macrophages. Animal studies have shown that peripheral blood monocytes from diabetic mice also exhibit a similar senescent phenotype and an elevated level of pro-inflammatory and pro-angiogenic factors.^47^ These findings may explain why local inflammation remains difficult to resolve during the proliferative diabetic retinopathy (PDR) stage, even when a partial transformation to an M2-like fibrosis or repair phenotype occurs.^48^^,^^49^ By continuously releasing SASP factors such as CXCL, TNF-α and IL-1β, senescent macrophages exacerbate neovascular leakage, extracellular matrix degradation, and fibrosis, creating a feedback loop that perpetuates the inflammatory state. Senescent microglia and infiltrating macrophages are not merely passive bystanders in the local microenvironment; rather, through the release of abundant EVs laden with miRNAs and inflammatory proteins, they directly induce astrocytes to enter a senescent state (as evidenced by p16/p21 upregulation) and enhance their SASP secretion.^44^ Meanwhile, senescent macrophages continuously recruit peripheral monocytes via the CXCL chemokine axis, generating an immune cascade amplification effect characterized by local senescence–sustained recruitment–de novo senescence.^47^ This mechanism explains why, at the PDR stage, local inflammation remains refractory to effective resolution even when a subset of macrophages convert to an M2-like reparative phenotype.^48^^,^^49^ Research in this area is primarily based on animal models and in vitro experiments, with direct evidence from human DR tissues remaining scarce.

### 3.5 Retinal ganglion cell senescence and early neurodegeneration

Damage to RGCs is a key cause of visual dysfunction in the early stage of diabetes.^32^ In vitro experiments have shown that under hyperglycemic conditions, the expression of the anti-senescence protein senescence marker protein 30 in RGCs is inhibited, leading to the accumulation of ROS and enhanced phosphorylation of NF-κB p65. This is accompanied by increased SASP factor secretion and downregulation of glutathione peroxidase and superoxide dismutase.^50^ Animal studies have shown that diabetes induces cellular senescence in RGCs and activates the SASP inflammatory pathway, representing a key mechanism underlying retinal neurodegeneration. Oxidative stress and cellular senescence act synergistically to impair both the structure and function of RGCs.^51^ Therefore, neuronal senescence leads to early visual function decline, providing support for “DR is not a simple vascular disease.” However, the above conclusions are primarily based on in vitro cell models; direct evidence of RGC senescence in diabetic animal models and retinas of DR patients requires further validation.

### 3.6 Crosstalk within the NVU: senescence propagation and synergistic damage

The retinal NVU, composed of vascular ECs, pericytes, basement membrane, glial cells (Müller cells and astrocytes), and neurons (primarily RGCs), relies on intricate intercellular communication and metabolic coupling for its integrity. In DR, senescence is not a cell-autonomous isolated event but rather propagates in a “contagious” manner within the NVU through SASP-mediated paracrine effects, forming a multi-cellular synergistic injury network.^5^^,^^8^

Bidirectional crosstalk between the vascular and neural compartments has been increasingly recognized. Senescent ECs secrete pro-inflammatory and pro-angiogenic SASP factors, including VEGF, IL-6, and ET-1, which can compromise pericyte coverage, increase vascular permeability, and destabilize the capillary network.^5^^,^^25^ In turn, pericyte senescence, characterized by upregulation of p16^INK4a^, p21, and SASP factors such as TGF-β1 and VEGF, reduces the supportive signals essential for endothelial survival and barrier maintenance.^52^ This vascular-derived senescent milieu may contribute to secondary injury in adjacent neural elements, consistent with the emerging view that DR involves early neurodegenerative changes alongside microvascular pathology.^32^^,^^42^

Glial cells, particularly microglia and astrocytes, function as central amplifiers of senescence signals within the NVU. In the diabetic retina, microglia undergo both activation and senescence-related transcriptional changes, releasing IL-1β, TNF-α, and EVs containing pro-senescence molecules that can induce astrocyte senescence and disrupt the glial support network.^42^^,^^44^ Senescent macrophages recruited from the circulation further perpetuate this inflammatory cascade by secreting SASP factors that promote neovascular leakage and extracellular matrix remodeling.^47^^,^^48^ The resulting glial dysfunction compromises neuronal survival and synaptic integrity, contributing to retinal neurodegeneration.^42^^,^^49^

Single-cell transcriptomic analyses have provided direct evidence for the coordinated activation of senescence pathways across NVU cell types. In db/db mouse retinas, senescence-associated transcripts including *Cdkn2a*/p16^INK4a^ and multiple SASP components show co-expression patterns across ECs, pericytes, and glial populations, suggesting that senescence signals are synchronously engaged within the NVU rather than emerging independently in each cell type.^5^^,^^14^ These observations support the concept that DR is fundamentally a disease of the entire NVU, wherein senescence propagates between cell types through paracrine SASP signaling, creating a self-perpetuating cycle of vascular dysfunction, glial inflammation, and neuronal damage.^8^^,^^25^^,^^44^

Collectively, these findings indicate that therapeutic strategies aimed solely at individual cell types may be insufficient; rather, interventions that disrupt senescence signal transmission across the NVU (whether by eliminating senescent cells, neutralizing SASP factors, or targeting key signaling nodes such as cGAS-STING) may offer greater potential for halting disease progression. However, the majority of current evidence is derived from animal models and in vitro systems; direct validation of NVU-wide senescence crosstalk in human DR tissues remains a critical priority for future investigation.

## 4 Molecular mechanisms driving retinal cell senescence in DR

### 4.1 Oxidative stress, DNA damage and telomere dysfunction

Mitochondrial dysfunction and NADPH oxidase (particularly NOX4) are primary sources of ROS accumulation in the diabetic retina, initiating a cascade of macromolecular damage that drives cellular senescence^20^^,^^50^ (shown in Fig. 2). In vitro experiments have demonstrated that excessive ROS triggers DNA damage response (DDR), primarily characterized by an increase in γH2AX/53BP1 damage foci and oxidative DNA modifications, such as 8-OHdG. This activates the p53/p21 pathway, leading to irreversible cell cycle arrest and the development of a senescent phenotype.^16^^,^^17^^,^^20^^,^^23^^,^^30^ In addition to affecting nuclear DNA, excessive ROS can also significantly damage mitochondrial DNA (mtDNA). In vitro studies have confirmed that oxidative damage and subsequent mtDNA leakage activate the cGAS-STING pathway.^53^ Activated STING then enhances the expression of inflammatory cytokines and VEGF through the NF-κB and HIF-1α pathways, further promoting cell senescence and SASP secretion.^54^ Recent in vivo experiments further confirmed that the level of mtDNA in the retinal cytoplasm of diabetic mice increased, and the cGAS-STING signaling axis was significantly activated. Conditional knockout of STING or use of STING inhibitors can significantly inhibit NF-κB-mediated inflammatory response and HIF-1α/VEGF-driven vascular leakage, indicating that this inflammatory amplification loop also plays an important role in the progression of DR in vivo.^14^^,^^21^^,^^24^^,^^31^^,^^47^^,^^55^ These findings collectively establish the mtDNA–cGAS–STING axis as a critical in vivo amplifier of senescence-associated inflammation in DR.

**Fig. 2:**
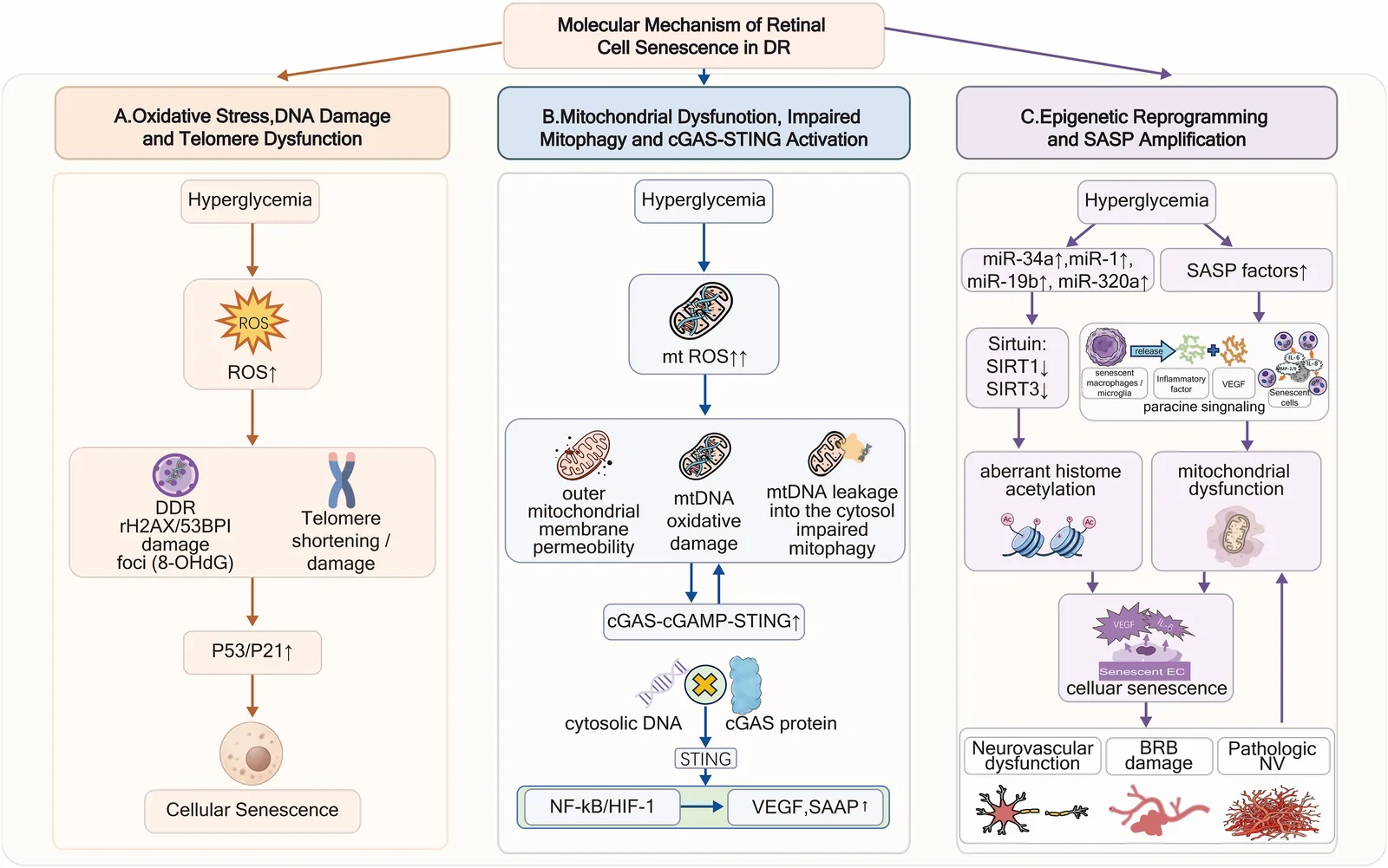
Molecular mechanism of retinal cell senescence in diabetic retinopathy. A. Oxidative Stress, DNA Damage and Telomere Dysfunction. B. Mitochondrial Dysfunction, Impaired Mitophagy and cGAS-STING Activation. C. Epigenetic Reprogramming and SASP Amplification.

With respect to telomere dysfunction, clinical data suggest that the shortening of telomere length in peripheral blood leukocytes is associated with an increased risk of DR, but the evidence of local telomere dysfunction in the retina and its direct activation of the p53/p21 aging pathway is still very limited, which has become a key link to be filled in this pathway. 56, 57, 58 The core links of the oxidative stress–DDR–senescence–STING inflammatory amplification pathway, while robustly established in vitro and increasingly supported by in vivo data, still require definitive validation in human retinal tissues to fully confirm their relevance to DR pathogenesis.

### 4.2 Mitochondrial dysfunction and impaired mitophagy

Mitophagy is a crucial mechanism for removing damaged mitochondria. In the early stages of DR, autophagy shows a transient compensatory increase (shown in Fig. 2). However, chronic hyperglycemia drives excessive mtROS production, which directly damages mitochondrial membrane integrity and mtDNA, overwhelming the autophagic clearance capacity.^59^^,^^60^ In vitro and in vivo studies have shown that prolonged hyperglycemia activates the mTOR pathway, inhibiting PINK1/Parkin-LC3B-mediated mitochondrial autophagic flux,^29^^,^^37^ leading to the accumulation of damaged mtDNA. This, in turn, activates the STING-VEGF signaling axis, further promoting cell senescence^15^^,^^40^(see detailed mechanistic dissection in Section 4.5). At the same time, while AMPK is upregulated in hyperglycemia, it is unable to effectively restore the autophagy-lysosomal fusion process. In fact, mechanistic studies suggest that AMPK may accelerate the premature senescence process by upregulating p16^INK4a30^. Transcriptomic data further reveal that the expression of autophagy-related genes, such as HIF1A and DRAM1, is significantly altered, supporting the close relationship between autophagy imbalance and the SASP phenotype.^30^

Critically, the relationship between mitophagy impairment and inflammation is bidirectional. SASP factors released by senescent cells (particularly IL-6 and TNF-α) can feedback to suppress SIRT3 and PINK1 expression, further exacerbating mitophagic blockade and creating a self-reinforcing “mitophagy failure-mtDNA release-STING activation-SASP-exacerbated mitophagy failure” vicious cycle^61^^,^^62^ (illustrated by the circular arrow in Fig. 2B). Of note, in vitro experiments have demonstrated that interfering with regulatory pathways of mitochondrial homeostasis can significantly delay the onset of the senescence phenotype. For instance, restoring SIRT3 level enhances mitochondrial antioxidant capacity and re-establishes autophagic flux,^25^ while downregulating mitochondrial membrane protein TIN2 reduces mTOR overactivation and restores mitochondrial autophagy, thereby improving the cellular functional phenotype.^37^ These findings demonstrate that the “autophagy-mitochondria-inflammation” damage cycle is a critical link in the process of cellular senescence in DR. The above conclusions are primarily derived from in vitro cell models; while the downstream STING-mediated inflammatory axis has been validated in animal models,^55^^,^^63^ definitive confirmation of the complete mitophagy–inflammation feedback loop in human DR tissues remains an important priority for future investigation.

### 4.3 Epigenetic reprogramming: miRNAs, Sirtuins and post-translational modifications

The diabetic microenvironment significantly alters the epigenetic landscape of retinal cells, with the miRNA network and sirtuin family proteins acting as key regulatory nodes (shown in Fig. 2). Hyperglycemia upregulates the expression of miR-34a, miR-1, miR-19b, and miR-320a, which directly suppress the expression of SIRT1 and SIRT3. This suppression leads to the abnormal accumulation of histone acetylation marks (H3K9ac and H4K16ac), disrupts redox homeostasis, impairs mitochondrial biogenesis, and collectively promotes cellular senescence, along with the activation of inflammation.^20^^,^^25^^,^^28^ Additionally, in vitro experiments have demonstrated that SIRT1 expression is significantly impaired under nutrient deprivation, further activating p16^INK4a^ and stabilizing the senescent phenotype.^20^^,^^38^ In terms of protein homeostasis, mechanistic studies have revealed that high glucose activates the p53-UBE2L6 signaling axis, promoting the ubiquitination and degradation of FoxO3a, weakening its key role in maintaining antioxidant defenses, energy homeostasis, and longevity regulation, ultimately accelerating RPE senescence.^21^ Therefore, after hyperglycemia-induced epigenetic damage, these alterations become more deeply involved in cell senescence and homeostasis, thereby exacerbating the progression of DR. Current evidence for epigenetic regulation is derived predominantly from in vitro studies, with direct validation in diabetic retinal tissues remaining limited.

### 4.4 Inflammatory signaling and SASP amplification

The SASP primarily consists of pro-inflammatory factors (IL-6, IL-8, IL-1β, TNF-α, and IFN-β), matrix remodeling enzymes (MMP-2/9), and growth factors (VEGF and TGF-β), all of which are closely linked to the pathology of DR (shown in Fig. 2). These factors not only sustain the senescent state of their producing cells but also propagate senescence signals to neighboring cells via paracrine pathways.^15^, ^16^, ^17^, ^18^, ^19^ In vitro studies have shown that SASP factors secreted by ECs, such as ET-1, VEGF, and fibronectin, can influence pericytes, glial cells, and adjacent ECs, thereby inducing secondary senescence and disrupting the integrity of the BRB.^25^ Similarly, in vitro experiments have demonstrated that SASP factors in RPE cells, such as IL-6 and IL-1β, activate the NF-κB signaling pathway, continuously enhancing VEGF expression. This process links BRB damage, inflammatory amplification, and pathological angiogenesis.^40^ It is important to note that secretory vesicles serve as a medium for the dissemination of senescence-related signals. Senescent cells release EVs carrying pro-inflammatory miRNAs and proteins that can be taken up by neighboring cells, thereby transferring senescence and amplifying the SASP response across the local microenvironment.^18^^,^^64^ This EV-mediated paracrine propagation further reinforces the spread of inflammation beyond the initially affected cell types.

Crucially, the relationship between SASP and mitochondrial dysfunction is bidirectional. While mitochondrial damage drives SASP production, SASP factors in turn feed back to exacerbate mitochondrial impairment: pro-inflammatory cytokines such as IL-6 and TNF-α suppress the expression of SIRT3 and PINK1, thereby inhibiting mitophagy and promoting the accumulation of dysfunctional mitochondria.^25^^,^^35^^,^^40^ This feedforward loop—whereby SASP reinforces mitochondrial damage and mitochondrial damage fuels further SASP secretion—constitutes a self-perpetuating inflammatory cycle that sustains chronic senescence and tissue dysfunction in the diabetic retina.

In summary, the SASP functions as both a consequence and a driver of cellular senescence, propagating damage across cell types via paracrine and EV-mediated pathways while simultaneously reinforcing mitochondrial dysfunction through feedback mechanisms. This integrated inflammatory network provides multiple nodes, ranging from SASP factor neutralization to EV inhibition and SIRT3 restoration, that represent promising druggable targets for disrupting the senescence–inflammation vicious cycle in DR.

### 4.5 Mitochondrial ROS, mtDNA leakage, and the cGAS-STING–SASP inflammatory feedforward loop

Mitochondria are not only the primary source of ROS but also a critical platform for the initiation of inflammatory signaling. In vitro studies have shown that under sustained high-glucose stimulation, the activity of mitochondrial electron transport chain complexes I and III is impaired, leading to a burst of mtROS. Excessive mtROS directly oxidizes cardiolipin on the mitochondrial membrane, increasing outer mitochondrial membrane permeability, and simultaneously induces mtDNA oxidative damage (8-OHdG formation) and fragmentation.^65^, ^66^, ^67^, ^68^ When mitophagy is blocked, damaged mtDNA leaks into the cytosol through the mitochondrial permeability transition pore or BAX/BAK channels, serving as a potent damage-associated molecular pattern.^69^, ^70^, ^71^

Mechanistic studies have demonstrated that cytosolic mtDNA is recognized by cGAS, which catalyzes the generation of the second messenger cGAMP, subsequently activating the endoplasmic reticulum adaptor protein STING. Activated STING then phosphorylates TBK1 and IRF3, inducing type I interferon (IFN-β) expression, while simultaneously activating the NF-κB signaling axis through the IKK complex, significantly upregulating the transcription of SASP factors including IL-6, TNF-α, and VEGF.^72^, ^73^, ^74^, ^75^ This already constitutes an inflammatory amplification pathway; more critically, these extracellular SASP factors (IL-6) act on adjacent cell membrane receptors through autocrine or paracrine pathways, further activating JAK/STAT and MAPK signaling, which in turn suppresses SIRT3 and PINK1 expression, thereby exacerbating mitophagic impairment and leading to further accumulation of damaged mitochondria and mtDNA leakage.^76^, ^77^, ^78^, ^79^

This constitutes a positive feedback cycle wherein high glucose-induced mtROS triggers mtDNA escape and cGAS-STING activation, resulting in SASP elevation. The ensuing suppression of mitophagy exacerbates mtDNA leakage, thereby closing the loop and reinforcing the pathological cascade (illustrated by the circular arrow in Fig. 2B). The existence of this loop means that even if the initial hyperglycemic stimulus is alleviated, retinal cells may maintain a sustained state of senescence and inflammation through this self-perpetuating mechanism. This finding provides a solid theoretical basis for targeting the cGAS-STING pathway or interrupting SASP signaling to break the inflammatory vicious cycle.^55^^,^^63^ The proposal of this positive feedback loop is based primarily on in vitro mechanistic studies, and its holistic validation in animal models is still in its infancy.

## 5 Senescence-targeted therapeutic strategies for diabetic retinopathy

### 5.1 Broad-spectrum senolytics targeting survival pathways

Senolytics are a class of drugs that selectively induce the death of senescent cells by targeting and inhibiting the specific anti-apoptotic signals, such as BCL-2, BCL-xL, PI3K/Akt, and STAT3. Animal studies have shown that clearing senescent cells in the OIR model reduces pathological neovascularization, restores vascular homeostasis, and improves retinal function.^8^^,^^12^^,^^80^ Among BCL-xL-targeted senolytics, the preclinical small molecule UBX1967 selectively eliminates senescent retinal vascular ECs, potently suppressing pathological angiogenesis and facilitating vascular remodeling5. Its clinical derivative UBX1325 (foselutoclax) has completed Phase 2 randomized trials in patients with DME; a single intravitreal injection achieved durable visual benefit over 48 weeks, marking the first human clinical proof-of-concept for senolytic therapy in diabetic retinal complications.^5^^,^^81^

In vitro studies in pulmonary fibroblasts confirm nintedanib exerts senolytic activity via STAT3 suppression; however, its senotherapeutic effects in high-glucose retinal cell models remain to be fully characterized.^82^^,^^83^ Other senolytics, such as dasatinib and quercetin (D + Q), fisetin and navitoclax (ABT-263), display robust senolytic effects across multiple systemic tissue senescence models and hold translational potential for DR.^84^^,^^85^ However, the clinical application of these senolytics is limited by the blood-retinal barrier, as systemic administration often leads to insufficient local drug concentration in the eye and off-target effects.^83^

Collectively, nearly all efficacy evidence for senolytics in DR stems from preclinical animal models, and robust clinical data specifically targeting proliferative diabetic retinopathy remain largely absent (only early-phase clinical outcomes for DME have been reported). Future translational research must address ocular delivery limitations via intravitreal injection strategies or targeted nanocarrier sustained-release platforms. Parallel development of retina-specific senescence biomarkers will also be critical to stratify patient populations most likely to respond to senolytic intervention.

### 5.2 Senomorphics and SASP modulators

Senomorphic agents alleviate senescent phenotypes by inhibiting SASP secretion or modulating senescence-related signaling pathways, without directly inducing cell death. These agents primarily include sirtuin activators and metabolic regulation drugs. Among them, the sirtuin family represents core upstream regulatory nodes governing mitochondrial homeostasis and senescence. In vitro high-glucose retinal cell experiments confirm that resveratrol activates SIRT1 and indirectly boosts mitochondrial SIRT3 activity, stabilizes mitochondrial membrane potential and oxidative phosphorylation, mitigates mtROS overproduction and suppresses downstream SASP secretion; its regulatory effects on SIRT4 and SIRT5 remain poorly characterized in retinal senescence models.^25^

In vitro studies in human retinal ECs demonstrate that GLP-1 receptor (GLP-1R) agonists upregulate SIRT1, repress the pro-senescent p53/p21 cascade, and maintain telomere homeostasis, thereby attenuating hyperglycemia-triggered premature endothelial senescence.^20^^,^^26^^,^^27^ The topical glucocorticoid fluorometholone activates Akt signaling, restores telomerase activity and upregulates visual cycle-related proteins to block SASP induction under high-glucose stress.^17^ Curcumin exerts senomorphic effects via context-dependent Akt modulation and broad antioxidant pathways, yet its regulation of retinal visual cycle machinery remains unvalidated in DR models.^19^

In vitro retinal studies confirm that metformin, a classic metabolic regulator, represses SASP cytokine expression and remodels inflammatory microenvironments via AMPK activation.^86^ SGLT2 inhibitors and low-dose mTOR inhibitors (rapamycin) also function as potent senomorphics by blunting NF-κB-mediated SASP transcription in diabetic retinal preclinical models, although direct evidence in retinal cells remains limited.^37^^,^^87^ Moreover, stem cell-derived exosomes strengthen cellular antioxidant defenses through the PI3K/Akt-Nrf2 axis, effectively blocking paracrine “senescence diffusion” and exerting multi-stage retinal protective effects across all DR stages.^18^

Notably, most mechanistic evidence for natural polyphenol senomorphics and stem cell exosomes derives solely from in vitro retinal cell culture systems. While GLP-1R agonists, metformin and fluorometholone have yielded partial in vivo protective outcomes in diabetic rodent DR models, long-term systemic safety profiles, intraocular delivery efficiency and definitive therapeutic efficacy in human DR patients remain largely unconfirmed. Future translational work requires systematic long-term animal studies and well-designed clinical trials to advance senomorphic therapies for diabetic retinal complications.

### 5.3 Precision delivery and combination therapeutic strategies

Single-targeted agents often fail to address the multi-cellular, multi-pathway senescence network in DR, making combination therapy an important avenue of exploration. Animal studies have shown that combining senolytics (ABT-263) with anti-VEGF therapy may simultaneously eliminate senescent cells and suppress aberrant angiogenesis.^88^, ^89^, ^90^, ^91^ Similarly, combining metformin with GLP-1 receptor agonists could theoretically yield additive metabolic and anti-inflammatory benefits, although synergistic senoprotective effects in retinal tissues remain to be validated.^92^^,^^93^ Furthermore, combining senolytics with antioxidants such as MitoQ may more effectively disrupt the senescence–inflammation–mitochondrial damage vicious cycle.^94^^,^^95^

However, posterior segment drug delivery remains a major bottleneck. Systemic administration is limited by the blood-retinal barrier, resulting in subtherapeutic ocular drug concentrations.^96^, ^97^, ^98^ Precision delivery platforms—including surface-modified nanoparticles, injectable thermosensitive hydrogels, and sustained-release intraocular implants—are under active preclinical and early clinical investigation to address these challenges.^99^, ^100^, ^101^, ^102^, ^103^, ^104^, ^105^ In principle, future “theranostic” nanoprobes incorporating senescence-specific biomarkers could enable localized detection and elimination of senescent cells, though this approach remains speculative.

### 5.4 Clinical translation challenges and future directions

Despite the promising preclinical prospects of senescence-targeting therapies, multiple critical obstacles remain for their clinical translation in DR.

First, bioavailability and drug delivery challenges. Current senolytics, including dasatinib and navitoclax, are primarily administered systemically. For BCL-xL-targeted senolytics (navitoclax, dasatinib combination), reaching therapeutic intraretinal concentrations via systemic oral dosing requires dose escalation, which exacerbates on-target adverse effects including thrombocytopenia and myelosuppression. However, the BRB substantially restricts the penetration of circulating drugs into the retina, often resulting in inadequate drug exposure at the target site. Achieving therapeutic intraretinal concentrations may therefore require increased systemic dosing, which could exacerbate dose-dependent adverse effects such as myelosuppression and thrombocytopenia. Consequently, the development of localized ocular delivery strategies has become an important direction for senolytic-based therapies.^106^, ^107^, ^108^ Topical eye drops cannot effectively penetrate the cornea and lens to reach the retina, while intravitreal injections carry risks of infection, hemorrhage, and elevated intraocular pressure and require repeated administration with poor patient compliance.

Second, off-target toxicity limits clinical application. Navitoclax (ABT-263), a representative senolytic agent targeting BCL-2 family proteins, induces potent on-target thrombocytopenia through inhibition of BCL-xL, a critical survival factor in human platelets, which has been consistently identified as a dose-limiting toxicity in multiple phase I/II clinical trials.^109^^,^^110^ Although the preclinical analog UBX1967 exhibited favorable local safety in OIR rodent models, its clinical derivative UBX1325 (foselutoclax) only completed 48-week Phase 2 DME trials; its long-term retinal neurotoxicity remains insufficiently characterized.^81^^,^^111^ Notably, intravitreal delivery of the optimized senolytic prodrug UBX1325 circumvents systemic hematologic toxicity, as validated in Phase 2 DME human trials, highlighting the critical value of localized ocular delivery strategies.^110^

Third, the lack of reliable senescence biomarkers for patient stratification and efficacy monitoring. Current assessment of senescence burden relies on invasive retinal biopsy or tissue SA-β-gal staining, which is not clinically feasible. Although some clinical studies have attempted to detect SASP factors (IL-6, VEGF) in aqueous humor or vitreous as indirect indicators,^48^^,^^49^these factors lack senescence specificity and are significantly influenced by systemic metabolic status. The development of non-invasive “senescence burden imaging” technologies based on optical coherence tomography (OCT), fluorescence lifetime imaging or AI retinal aging clocks, or the establishment of liquid biopsy systems based on peripheral blood cell-free p16 promoter methylation and ocular extracellular vesicle signatures, represents a promising but still exploratory direction.^112^

Fourth, clinical trial endpoint design remains controversial. The clinical hard endpoints of DR (visual acuity decline, neovascularization) typically require years to manifest, whereas the early benefits of senescent cell clearance are more apparent in the attenuation of microvascular leakage and glial responses. How to design surrogate endpoints that are both scientifically persuasive and under active exploration in clinical trial design (OCT angiography parameters, microaneurysm regression rate) represents a key challenge for advancing senolytics into Phase III clinical trials.^7^

## 6 Conclusion

In diabetic retinopathy, the convergence of oxidative stress, epigenetic modifications, and impaired mitophagy drives cell-type-specific senescence across ECs, pericytes, retinal pigment epithelium, glial cells, and neurons. These senescent cells do not act in isolation; rather, they engage in reciprocal crosstalk within the NVU, propagating damage via paracrine SASP signaling. This establishes a self-perpetuating senescence-inflammation positive feedback loop that underpins chronic vascular leakage, pathological neovascularization, and neurodegeneration, positioning cellular senescence as an upstream central contributor and amplifier of DR pathology.

Despite these mechanistic insights, the clinical translation of senotherapies faces substantial hurdles. The blood-retinal barrier severely restricts the ocular bioavailability of systemically administered agents, while the lack of validated, non-invasive senescence biomarkers precludes patient stratification and real-time efficacy monitoring. Moreover, broad-spectrum senolytics such as navitoclax carry dose-limiting off-target toxicities, and their long-term retinal safety data remain limited.

To bridge this translational gap, we advocate for three priority research directions: first, the development of intraocular sustained-release formulations (hydrogels or nanoparticle-based carriers) to achieve sustained therapeutic concentrations; second, the identification of cell-type-specific senolytic targets to minimize systemic adverse effects; and third, the initiation of prospective cohort studies integrating multi-omics and advanced imaging to establish reliable senescence biomarkers for patient selection. Combinatorial regimens integrating senolytics with anti-VEGF agents or senomorphics may further enhance efficacy while mitigating monotherapy limitations. Overcoming these obstacles will be essential to translate senescence-targeting strategies into viable personalized therapies for early-stage DR and retinal repair.

## Study approval

Not Applicable.

## Author contributions

The authors confirm contribution to the paper as follows: Conception: WL, JG, XL, LS, JC; Investigation: XZ; Formal Analysis: FY, MJ; Writing – original draft: XL, LS, JC; All authors reviewed the results and approved the final version of the manuscript.

## Declaration of generative AI use

During the preparation of this work, the authors used ChatGPT only for language polishing and grammar checking. The authors have reviewed and edited the content and take full responsibility for the final manuscript.

## Funding

This work was supported by 10.13039/501100001809National Natural Science Foundation of China (82471106) and Tianjin Key Medical Discipline Construction Project (TJYXZDXK-3-004A-2).

## Declaration of competing interest

The authors declare that they have no known competing financial interests or personal relationships that could have appeared to influence the work reported in this paper.
